# Supplementary material for: Quantitative Structure–Activity Relationships for the Flavonoid-Mediated Inhibition of P-Glycoprotein in KB/MDR1 Cells
Source: Molecules. 2019 Apr 27;24(9):1661. doi: 10.3390/molecules24091661 (PMC6539955; doi:10.3390/molecules24091661)
Supplement: Supplementary file 1 [file molecules-24-01661-s001.pdf]

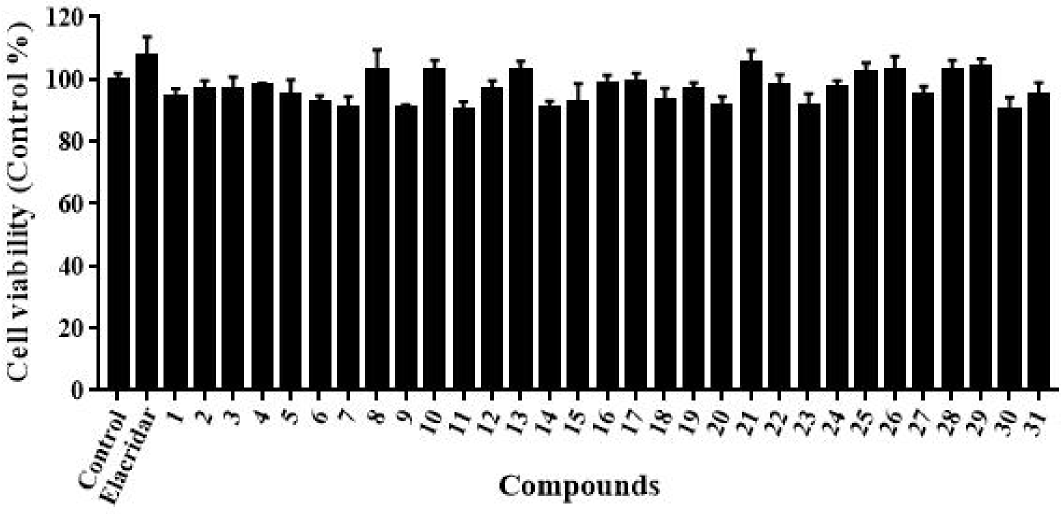

Figure 1S. The cytotoxicity of flavonoids and elacridar in KB/MDR1 cells.

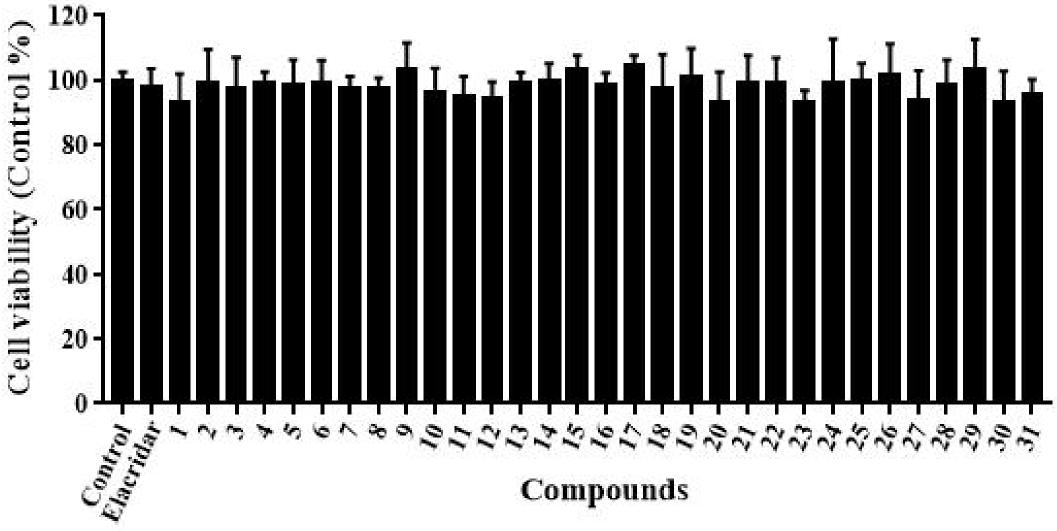

Figure 2S. The cytotoxicity of flavonoids and elacridar in KB cells.
